# Characterization of High and Low IFNG-Expressing Subgroups in Atopic Dermatitis

**DOI:** 10.3390/ijms25116158

**Published:** 2024-06-03

**Authors:** Sophia Wasserer, Manja Jargosch, Kristine E. Mayer, Jessica Eigemann, Theresa Raunegger, Görkem Aydin, Stefanie Eyerich, Tilo Biedermann, Kilian Eyerich, Felix Lauffer

**Affiliations:** 1 Department of Dermatology and Allergy, Technical University of Munich, 80802 Munich, Germany; sophia.wasserer@tum.de (S.W.);; 2 Center of Allergy & Environment (ZAUM), Technical University of Munich, Helmholtz Center Munich, 80802 Munich, Germany; 3 Department of Dermatology and Allergy, Medical Center, University of Freiburg, 79104 Freiburg, Germany

**Keywords:** atopic dermatitis, IFNG, IFN-γ, subgroups/endotypes, IL-4RA1, M1 macrophages, natural killer cells

## Abstract

Atopic dermatitis (AD) is one of the most common chronic inflammatory skin diseases, with an increasing number of targeted therapies available. While biologics to treat AD exclusively target the key cytokines of type 2 immunity, Janus kinase inhibitors target a broad variety of cytokines, including IFN-γ. To better stratify patients for optimal treatment outcomes, the identification and characterization of subgroups, especially with regard to their IFNG expression, is of great relevance, as the role of IFNG in AD has not yet been fully clarified. This study aims to define AD subgroups based on their lesional IFNG expression and to characterize them based on their gene expression, T cell secretome and clinical attributes. RNA from the lesional and non-lesional biopsies of 48 AD patients was analyzed by RNA sequencing. Based on IFNG gene expression and the release of IFN-γ by lesional T cells, this cohort was categorized into three IFNG groups (high, medium, and low) using unsupervised clustering. The low IFNG group showed features of extrinsic AD with a higher prevalence of atopic comorbidities and impaired epidermal lipid synthesis. In contrast, patients in the high IFNG group had a higher average age and an activation of additional pro-inflammatory pathways. On the cellular level, higher amounts of M1 macrophages and natural killer cell signaling were detected in the high IFNG group compared to the low IFNG group by a deconvolution algorithm. However, both groups shared a common dupilumab response gene signature, indicating that type 2 immunity is the dominant immune shift in both subgroups. In summary, high and low IFNG subgroups correspond to intrinsic and extrinsic AD classifications and might be considered in the future for evaluating therapeutic efficacy or non-responders.

## 1. Introduction

Atopic dermatitis (AD) is a chronic inflammatory skin disease with increasing prevalence worldwide. Its pathogenesis is based on an impaired skin barrier, a type 2-dominant immune response and a dysbiosis of the skin microbiome [1]. It is well accepted that AD is a heterogeneous disease, and several disease endotypes have been described in the literature [2]. Though numerous new treatment options have been approved over the last decade, there is still a proportion of approximately 30% of patients not reaching an optimal clinical response [3,4]. In this context, the role of IFN-γ in AD is currently gaining renewed interest due to the different modes of action of new treatments. While the anti-IL-4Rα antibody dupilumab and the anti-IL-13 antibodies tralokinumab and lebrikizumab exclusively target specific type 2 immune cytokine functions, Janus kinases 1/2 inhibitors modify the signal transduction of numerous cytokines, including IFN-γ as well as type 2 cytokines [1].

In the 1990s and 2000s, IFN-γ was a major focus of AD research. Numerous studies demonstrated the reduced capacity of peripheral T cells to produce IFN-γ and a reduced presence of IFN-γ in the affected skin of AD patients [5,6,7]. Therefore, IFN-γ was considered as a possible treatment option and several studies investigated the therapeutic administration of IFN-γ. Despite significant clinical improvement [8,9,10,11], recombinant human IFN-γ therapy has not become part of the conventional AD therapies [12].

To date, the role of IFN-γ in AD is still not fully understood. IFN-γ is produced by natural killer (NK) cells and T cells and signals via STAT1 and STAT4. Its main physiological task is the recognition of and defense against intracellular viruses and malignant cells [12,13,14]. IFN-γ prevents the differentiation of T-helper (Th) 2 cells, IL-4-induced IgE production and the expression of IL-4R on keratinocytes, while in return type 2 cytokines suppress Th1 polarization [7,15,16,17,18]. Several studies proved the potential of IFN-γ to contribute to an impaired skin barrier by downregulating tight junctions or ceramide synthesis. Furthermore, IFN-γ mediates Fas-dependent keratinocyte apoptosis and promotes inflammation, especially in the context of contact dermatitis [19,20,21,22]. Moreover, AD patients with low lesional IFN-γ levels are at higher risk of developing eczema herpeticum, a widespread herpes simplex infection of the skin [23,24]. While higher IFN-γ levels were detected in chronic AD lesions, it remains unclear whether a high IFN-γ expression in AD exhibits predominantly protective effects or if it rather contributes to chronicity and maintained inflammation in AD [25,26]. From a clinical perspective, it is therefore crucial to clarify whether there are certain subgroups of AD patients who would benefit from additional targeting of IFNG or, conversely, whether the inhibition of type 2 immunity alone is sufficient for the treatment of AD even in subgroups with high IFNG expression. Therefore, the aim of this work is to characterize high and low IFNG subgroups in AD with respect to their clinical characteristics, comorbidities, differences in gene expression and regulation of dupilumab-responsive genes.

## 2. Results

### 2.1. Transcriptome Analysis Reveals High, Medium and Low IFNG Subgroups of AD Correlating with Patients’ Ages

**Figure 1:**
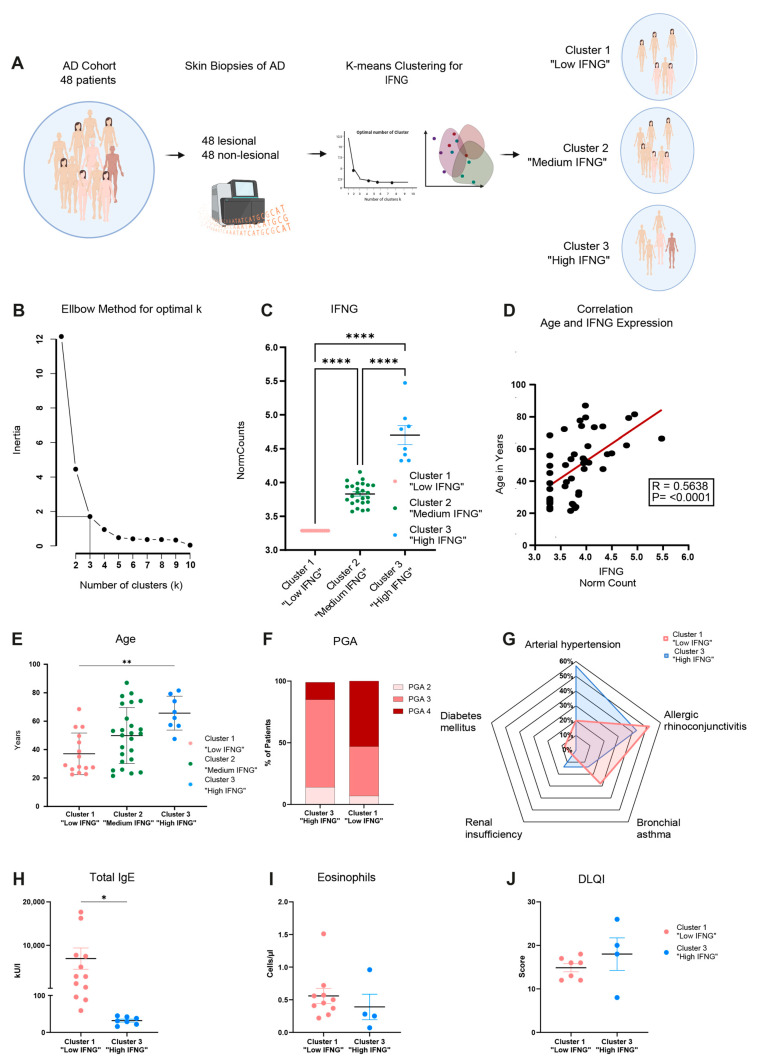
Transcriptome analysis reveals high, medium and low IFNG subgroups of AD correlating with patients’ ages. (**A**) Study design: lesional and non-lesional skin biopsies from 48 AD patients were collected, and their bulk RNA was sequenced and categorized into 3 IFNG subgroups based on their lesional IFNG expression by unsupervised clustering. (**B**) Elbow method identified k = 3 as the optimal number of IFNG clusters for the AD cohort. (**C**) Normalized IFNG gene counts in AD patients separated by low (*n* = 15), medium (*n* = 25) and high (*n* = 8) IFNG expression. (**D**) Pearson correlation of patient’s age and normalized IFNG gene expression for lesional AD biopsies (*n* = 48). (**E**) Age distribution within the low (*n* = 15), medium (*n* = 25) and high (*n* = 8) IFNG AD subgroups. (**F**) Physician Global Assessment (PGA) for evaluating the disease severity score of high and low IFNG subgroups. (**G**–**J**) Clinical and laboratory characterization of the low and high IFNG AD subgroups in relation to their co-morbidities ((**G**), radar plot), serum total IgE concentrations (**H**), number of blood eosinophils (**J**), and the patient’s dermatology quality of life index (DLQI, (**I**)). Group comparison was performed using one-way ANOVA test with multiple comparisons. The significance levels were defined as *p* ≤ 0.05 (*), *p* ≤ 0.01 (**) and *p* ≤ 0.0001 (****). AD = atopic dermatitis, PGA = Physician Global Assessment, DLQI = Dermatology Life Quality Index.

The transcriptome of, in total, 48 lesional and corresponding non-lesional skin biopsies of AD patients was analyzed by RNA sequencing. Based on lesional IFNG expression, the AD cohort was first categorized into IFNG subgroups by unsupervised clustering (Figure 1A). The optimal number of clusters (k = 3) was determined using the elbow method (Figure 1B). Subsequently, k-mean clustering with k = 3 identified 8 patients as cluster 3 “high IFNG” (IFNG mean-normalized counts = 4.7), 25 patients as cluster 2 “medium IFNG” (IFNG mean-normalized counts = 3.83) and 15 patients as cluster 1 “low IFNG” (IFNG mean-normalized counts = 3.29), showing significant (*p* ≤ 0.0001) differences in their expression of IFNG (Figure 1C). This characterization of the cohort showed there was a positive correlation (R = 0.5638, *p* ≤ 0.0001) of IFNG expression with patients’ ages (high IFNG group 66 ± 12 years, intermediate IFNG group 50 ± 20 years, low IFNG group 37 ± 15 years) (Figure 1D,E). A principal component analysis of the whole lesional skin transcriptome revealed minor separation between cluster 1 and cluster 2/cluster 3 (PCA1 = 15.01%) (Figure S1), which is why the following analysis focused on the differences between the “low IFNG” cluster 1 and “high IFNG” cluster 3. The analysis of cluster 2 “medium IFNG” is provided in the Supplementary Materials (Figures S2 and S3). To further characterize the high IFNG and the low IFNG cohort, the severity of the disease was evaluated using the Physician Global Assessment (PGA) score, ranging from 0 (clear) to 4 (very severe disease). More patients in the low IFNG cohort were severely affected than in the high IFNG cohort (PGA-4: 53% vs. 14%) (Figure 1F). Next, clinical and laboratory characterization revealed that the “low IFNG” cluster exhibited features of extrinsic AD with an increased presence of bronchial asthma (28% vs. 14%) and allergic rhino conjunctivitis (52% vs. 43%) (Figure 1G), as well as high serum IgE levels (6937 ± 2443 kU/I vs. 31.9 ± 3.9 kU/I) (Figure 1H) compared to the “high IFNG” cluster. In contrast, significantly more patients in the “high IFNG” cohort suffered from arterial hypertension (57% vs. 20%) and renal insufficiency (14% vs. 4%) compared to the “low IFNG” cluster (Figure 1G). In terms of the amount of blood eosinophils (Figure 1I) and the Dermatology Life Quality Index (DLQI) (Figure 1J), there were no significant differences between both clusters. In summary, the high IFNG subgroup is characterized by a higher age and exhibits an intrinsic AD endotype, while the low IFNG subgroup is characterized by a lower age and exhibits an extrinsic AD endotype.

### 2.2. Ex Vivo Secretome Analysis of Lesional T Cells of AD Patients Confirms High and Low IFN-γ Subgroups

**Figure 2:**
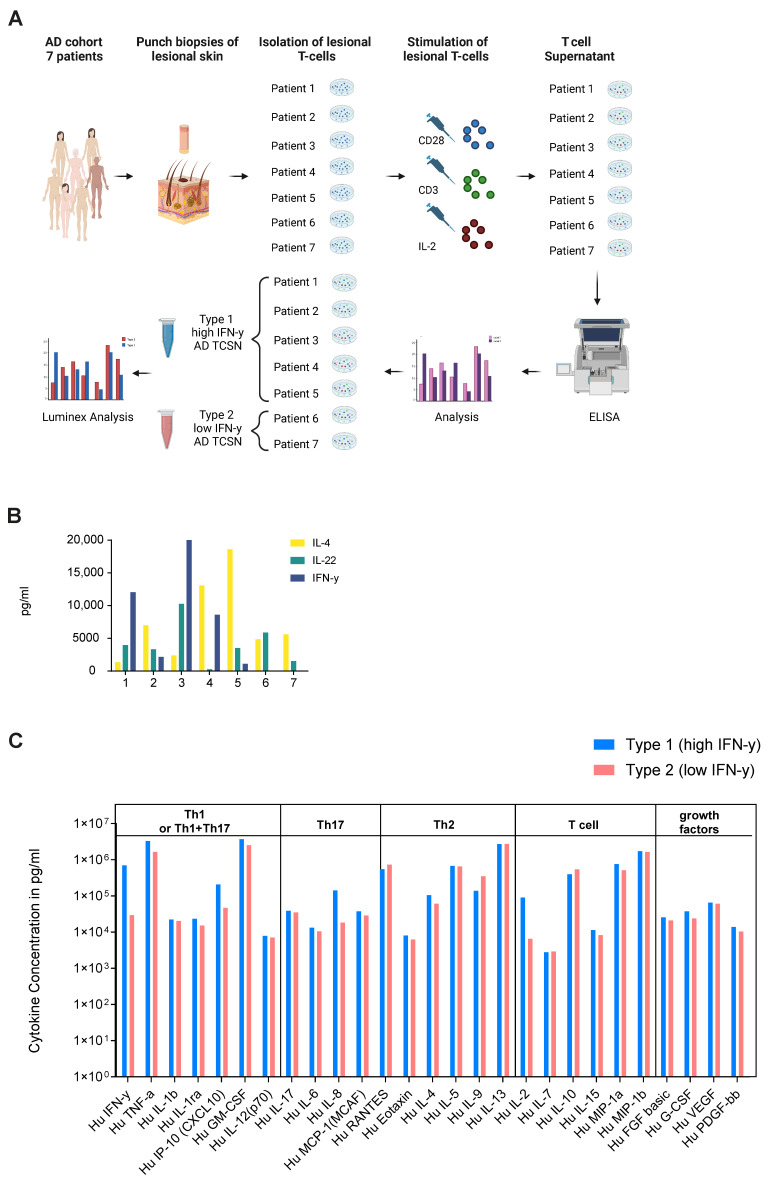
Ex vivo secretome analysis of the lesional T cells of AD patients confirms high and low IFN-γ subgroups. (**A**) Generation of lesional AD T cell supernatant (TCSN): Lesional T cells from the punch biopsies of 7 atopic dermatitis (AD) patients were isolated, expanded and stimulated to collect an AD TCSN. Subsequently, TCSNs were pooled into TCSNs with low (*n* = 2) and high (*n* = 5) IFN-γ contents based on their IFN-γ secretion and characterized by Luminex analysis. (**B**) Concentrations of IFN-γ, IL-4 and IL-22 in the AD TCSNs of individual patients, measured by ELISA. (**C**) Twenty-seven-plex Luminex analysis of the pooled high, type 1 and the low, type 2 IFN-γ AD TCSN.

To validate the results of the high and low IFNG AD subtypes identified in the transcriptome study, punch biopsies were collected from seven different AD patients to isolate their lesional T cells. After expansion and stimulation, the generated lesional T cell supernatant (TCSN) was characterized by ELISA for IL-4, IL-22 and IFN-γ (Figure 2A,B). Interestingly, T cells from two patients (Patient 6 and 7) did not produce IFN-γ. Subsequently, their TCSNs were pooled to generate a low IFN-γ- (patient 6 and 7) and high IFN-γ (patient 1–5)-containing AD TCSN. To further characterize the two TCSN groups, a Luminex analysis was performed as a bioplex assay for 27 chemokines and cytokines. Here, a higher amount of IFN-γ (701,328 vs. 29,585 pg/mL), TNF (3,313,489 vs. 1,640,775 pg/mL), IL-8 (141,764 vs. 18,399 pg/mL) and IL-2 (91,028 vs. 6583 pg/mL) was observed in the high compared to the low IFN-γ AD TCSN (Figure 2C). No significant differences were observed for the Th2 cytokines IL-4, IL-5 and IL-13. Thus, the ex vivo secretome analysis of lesional AD T cells could confirm the presence of high- and low-IFN-γ AD subgroups. However, both IFN-γ subgroups were defined by their expression and release of Th2 cytokines.

### 2.3. Low and High IFNG AD Subgroups Show Distinct Molecular Pathways with a Strong Activation of Type 1 Immunity in the High IFNG Group

**Figure 3:**
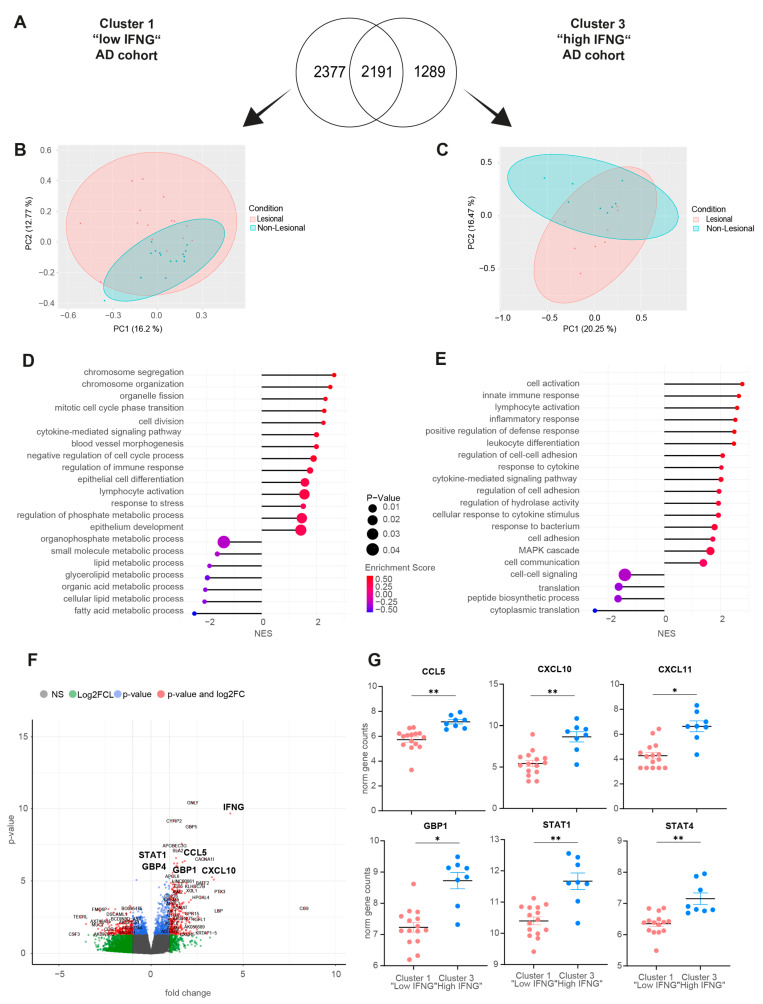
Low and high IFNG AD subgroups show distinct molecular pathways, with a strong activation of type 1 immunity in the high IFNG group. (**A**–**C**) Differentially expressed gene (DEG) analysis between lesional and non-lesional skin for the high and the low IFNG AD subgroups: In (**A**), the number of DEGs is visualized in a Venn diagram for both groups. In (**B**,**C**), the separation between lesional and non-lesional skin is shown within the low (**B**) and high (**C**) IFNG groups by principal component analysis. (**D**,**E**) GO term pathway analysis: enriched pathways within the low (**D**) and high (**E**) IFNG AD subgroups are shown. (**F**,**G**) DEG analysis of the high vs. low IFNG AD subgroups: DEGs are visualized in a volcano plot (**F**). Normalized gene counts for identified differentially regulated genes are shown for the low (*n* = 15) and high (*n* = 8) IFNG groups (**G**). Group comparison was performed using a *t*-test. The significance levels were defined as *p* ≤ 0.05 (*) and *p* ≤ 0.01 (**). DEG = differentially expressed gene, PC = principal component, NES = normalized enrichment score, NS = not significant, FC = fold change.

Next, the high and low IFNG AD subgroups were characterized by a transcriptome analysis. For this purpose, differentially expressed genes (DEGs) were calculated by comparing matched lesional and non-lesional skin biopsies. In total, the low IFNG group showed slightly more DEGs, with 4568, than the high IFNG group with 3480 DEGs, while 2191 of those genes were regulated in both clusters (Figure 3A). In contrast, the high IFNG group (PC1 = 20.25%, PC2 = 16.47%) showed a larger separation between the transcriptomes of lesional and non-lesional skin compared to the low IFNG group (PC1 = 16.20%, PC2 = 12.77%) (Figure 3B,C), indicating a stronger alteration of unaffected skin in extrinsic AD skin.

Next, a GO-term enrichment analysis was performed. Interestingly, in the low IFNG group, pathways related to cell cycle processes (e.g., chromosome segregation, mitotic cell cycle phase transition, cell division), immune responses (regulation of immune response, lymphocyte activation) and epithelium development were upregulated, while pathways associated with lipid metabolism (fatty and organic acid/cellular and glyerolipid/organophosphate metabolic process) were downregulated (Figure 3D). In contrast, the signature of the high IFNG group was dominated by reactions of the innate and adaptive immune system (e.g., innate immune response, lymphocyte activation, leukocyte differentiation), as well as the response to bacterium and a general inflammatory response (Figure 3E).

Finally, the DEGs between the transcriptome of lesional AD skin from the high and low IFNG groups were calculated to compare both groups at the gene level (Figure 3F). The volcano plot of the high vs. low IFNG groups revealed significantly upregulated genes in the high compared to low IFNG group; among them were the type I immune response genes *CXCL10* (*p* = 0.0051), *CXCL11* (*p* = 0.0122), *STAT4* (*p* = 0.0064) and *STAT1* (*p* = 0.0082), as well as the chemoattractant *CCL5* (*p* = 0.0051) and the interferon (IFN)-inducible GTPase *GPB4* (*p* = 0.00135), activators of the inflammasome (Figure 3F,G).

In summary, the transcriptome analysis of the high IFNG group showed a strong activation of type 1 immunity, including the innate immune system and viral and bacterial defense and Th1-related genes, whereas the low IFNG group showed a significant reduction in lipid metabolism.

### 2.4. Digital Cytometry Reveals Higher Amounts of M1 Macrophages, NK Cells and CD4 Memory T Cells in the High IFNG AD Subgroup

**Figure 4:**
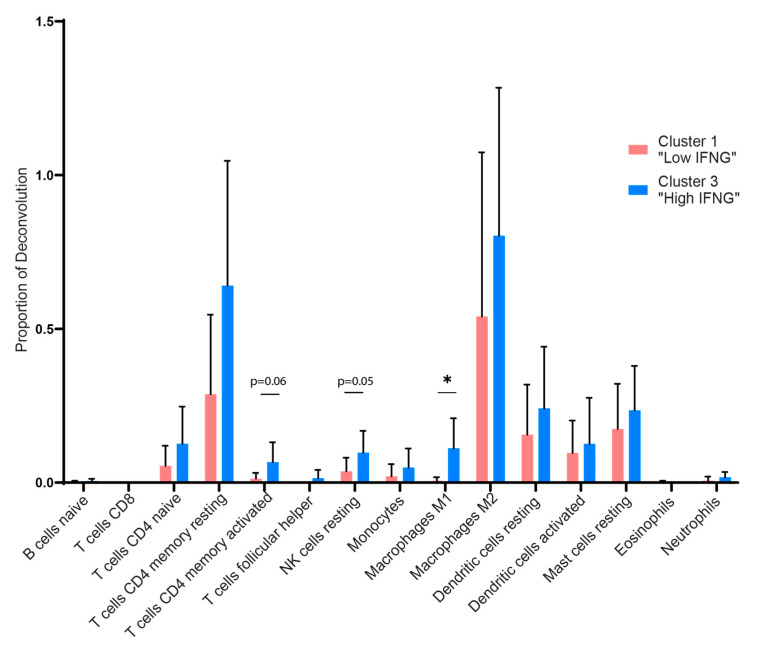
Deconvolution algorithm reveals higher amounts of immune cells such as M1 macrophages, NK cells and CD4 memory T cells in the high IFNG group. The CIBERSORT algorithm was used to perform a deconvolution with regard to the presence of immune cell populations in the low (red) and high (blue) IFNG AD subgroups. LM22, a gene matrix of 22 distinct human hematopoietic cell populations provided by CIBERSORT, was used as the signature matrix. The proportion of deconvolution is visualized for the individual immune cell populations. Group comparison was performed using a paired *t*-test. The significance level was defined as *p* ≤ 0.05 (*).

To further characterize and quantify differences regarding the immune cell subsets in the low and high IFNG AD subgroup, we performed an in silico cytometry analysis by applying the deconvolution algorithm CIBERSORTx. In accordance with the transcriptome and pathway analysis, a higher presence of immune cells was observed in the high IFNG group (Figure 4). In detail, the high IFNG group revealed a higher amount of the total estimated fraction of M1 polarized macrophages (*p* = 0.015) and a tendency towards more resting NK cells (*p* = 0.051), as well as CD4^+^-activated memory cells (*p* = 0.060), compared to the low IFNG group. There was no significant difference between CD8^+^ T cells, neutrophils, eosinophils, M2 macrophages and dendritic cells. This indicates that, apart from M1 macrophages and possibly NK cells, the cellular composition of lesional AD skin is similar between the two subgroups.

### 2.5. Gene Set Variation Analysis Shows a Higher Cardiovascular Risk, a More Pronounced Activation of the Immune System and a Higher Enrichment of Type 1-Associated Genes in the High IFNG AD Subgroup

**Figure 5:**
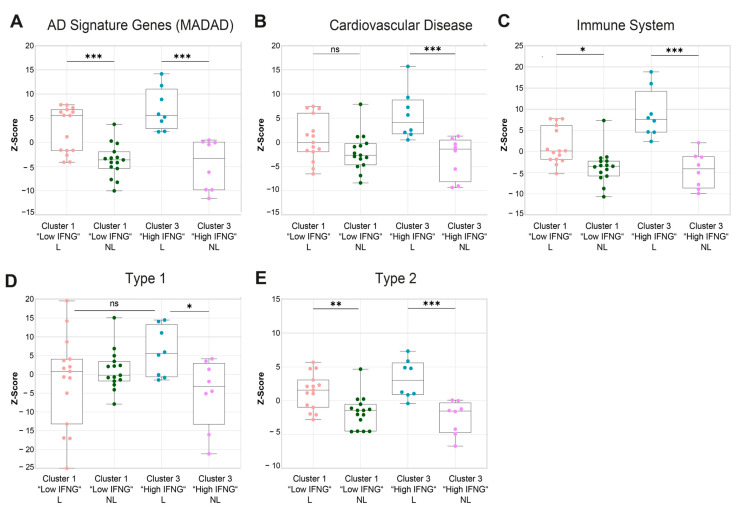
Gene set variation analysis shows a higher cardiovascular risk, a stronger activation of the immune system and a higher enrichment of type 1-associated genes in the high IFNG AD subgroup. (**A**–**E**) A gene set variation analysis (GSVA) was performed for different gene set collections associated with AD signature genes (MADAD, (**A**)), cardiovascular diseases (**B**) and the immune system (GO, (**C**)), as well as type 1/type 2 keratinocyte immune signatures (**D**,**E**), with lesional (L) and non-lesional (NL) low (*n* = 15) and high (*n* = 8) IFNG AD transcriptomes. The enrichment is visualized as Z-scores. Group comparison was performed for a mixed-effect analysis with Tukey’s multiple comparison test. The significance levels were defined as ns = non-significant, *p* ≤ 0.05 (*), *p* ≤ 0.01 (**), *p* ≤ 0.001 (***).

Next, a gene set variation analysis (GSVA) of publicly available gene sets associated with AD (MADAD) [27], cardiovascular diseases [28,29,30], the immune system [29,31] and type 1 and type 2 keratinocyte response genes [32] was performed, identifying further differences between the low and high IFNG AD subtypes, including in the analysis of lesional and non-lesional skin. As expected, the established gene signature for AD (meta-analysis-derived atopic dermatitis (MADAD)) was significantly enriched in the lesional skin biopsies of both the high (*p* = 0.0005) and low (*p* = 0.0004) IFNG groups, with a tendency towards greater enrichment in the high IFNG group (Figure 5A). Additionally, there was a significantly higher upregulation of genes associated with cardiovascular diseases (*p* = 0.0024, Figure 5B) and the immune system (*p* = 0.0001; Figure 5C) in the high compared to the low IFNG group. To further characterize the specific immune response patterns in both groups, signature gene sets were extracted from a microarray analysis of stimulated keratinocytes under type 1 and type 2 cytokine conditions, as described previously [32], and a GSVA was performed (Figure 5D,E). For both immune response patterns, the signature genes were highly enriched in lesional compared to non-lesional skin in both IFNG groups, except for type 1 genes in the low IFNG group (Figure 5D,E). Overall, the gene set for type 2-related genes showed the largest differences between lesional and non-lesional skin (Figure 5E). Consistent with previous findings, type 1 immune response genes were more enriched in the lesional skin of the high compared to the low IFNG AD subgroup, again suggesting an activation of the type 1 axis by IFN-γ (Figure 5D). Of note, there were no differences in type 2-related genes between the two IFNG groups, suggesting that, regardless of IFNG expression, type 2 signaling is the dominant common pathway.

### 2.6. High and Low IFNG AD Subgroups Share a Strong Common Core Signature with Dupilumab Response Genes

**Figure 6:**
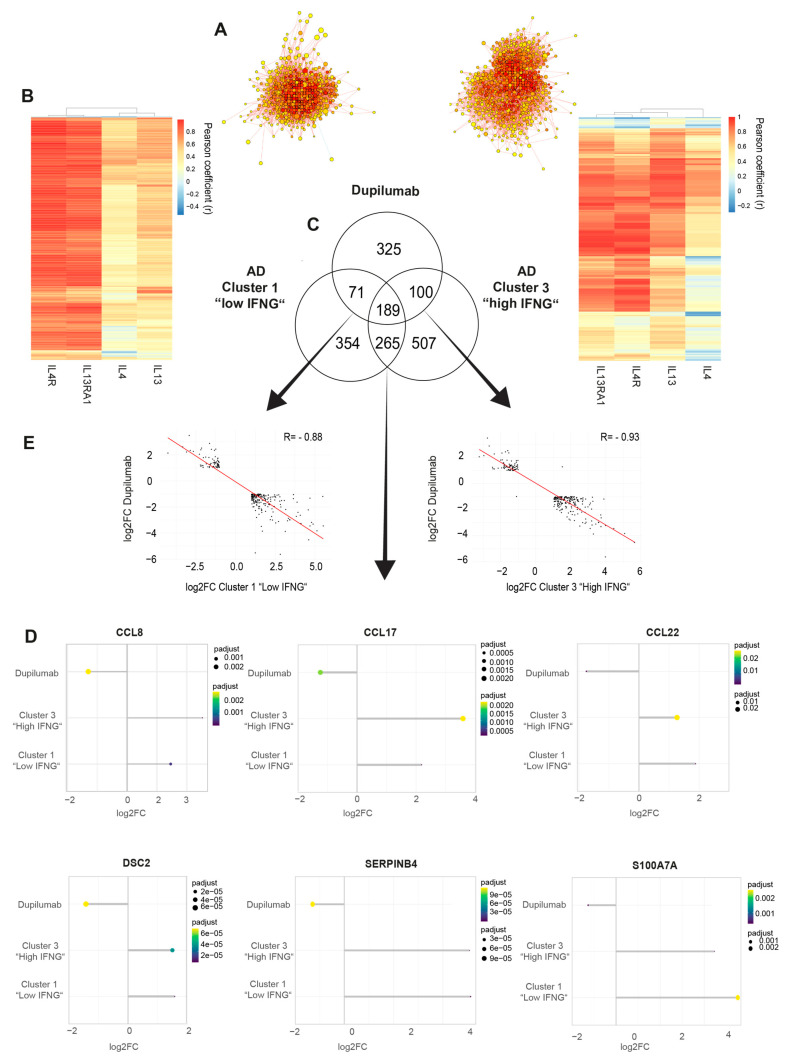
High and low IFNG AD subgroups share a strong common core signature with dupilumab response genes. (**A**) Centrality ranked genes were identified as core signature genes for the low (left) and high (right) IFNG AD subgroups by the INFORM consent algorithm and visualized in a gene interaction network. The red coloration of the genes indicates their heightened centrality and importance within the gene network. (**B**) Pearson correlation was performed between the (**A**) identified core signature genes for the low (left) and high (right) IFNG AD groups and the type 2-related genes *IL4R*, *IL13RA1*, *IL4* and *IL13*. (**C**–**E**) The DEGs (LS vs. NL) of core signature genes of the high (*n* = 1061) and low (*n* = 879) IFNG AD subgroups were compared to dupilumab response genes (*n* = 685, lesional skin on week 16 after dupilumab initiation vs. week 0) [33] and visualized in a Venn diagram (**C**). Log2FCs of the dupilumab response genes and low IFNG (left) or high IFNG (right) DEGs are displayed as scatter plots and linear regression was applied (R = −0.93 for the high IFNG group, R = −0.88 for the low IFNG group) (**D**). In total, 189 genes were commonly regulated by dupilumab and the high and low IFNG groups, including *CCL8*, *CCL17*, *CCL22*, *DSC2*, *SERPINB4* and *S100A7A*. Log2FC and p-adjusted values of these selected commonly regulated genes were visualized for the dupilumab response and low and high IFNG AD groups (**E**). DEG = differentially expressed gene, FC = fold change.

Finally, to gain further insights into the therapeutic relevance of the IFNG content in AD subgroups, the transcriptome of the low and high IFNG AD subgroups was compared to a dupilumab response signature (lesional skin at week 16 after dupilumab initiation vs. week 0), as previously described [33]. For this purpose, the most central genes of both groups were first determined by the INFORM consensus algorithm, as described previously [34], and combined into a core signature for the low (Figure 6A left) and high (Figure 6A right) IFNG AD groups. Next, the gene expressions of the identified core signature genes were correlated to the most important type 2-related genes: IL4R, IL13RA1, IL4 and IL13 (Figure 6B). The core signature genes of the low IFNG AD group (Figure 6B left) showed a strong positive correlation with IL4R and IL13RA1 which was less pronounced in the high IFNG AD group (Figure 6B right). The cytokines *IL4* and *IL13* exhibited a lower overall correlation.

Subsequently, the DEGs of lesional vs. non-lesional core signature genes were calculated for the high (*n* = 1061) and low (*n* = 879) IFNG AD groups and were compared with dupilumab response genes (*n* = 685) to characterize the gene response pattern of both AD groups with regard to dupilumab therapy (Figure 6C). Overall, the dupilumab signature was strongly regulated in both AD groups. A total of 52.5% (360 genes) of the dupilumab response genes were present in the DEGs of the low and/or high IFNG AD group, of which 189 DEGs were commonly regulated in all three groups. Interestingly, 59.3% of these commonly regulated genes were upregulated in the high and low IFNG AD groups and downregulated by dupilumab. Among those genes were driver genes of AD, such as *CCL8*, *CCL17* and *CCL22*, as well as *SERPINB4*, *S100A7A* and *DSC2* (Figure 6E). Conversely, only a total of 4.2% of these commonly regulated genes were downregulated in the high and low IFNG AD groups and upregulated by dupilumab, while 36.5% of all commonly regulated genes were regulated in the same direction.

Looking at the difference between low and high IFNG, 71 genes were only regulated by dupilumab and the low IFNG AD group (Figure 6C). All of them were regulated in opposite directions (Figure 6D left), and among them were *IL13RA2* (Dupi DOWN, low IFNG group UP) and *ELOVL3* (Dupi DOWN, low IFNG group UP). The same applies to the genes exclusively shared between dupilumab and the high IFNG AD group. Again, 98% of the 100 genes were regulated in opposite directions (Figure 6C,D right), including *STAT1* (Dupi DOWN, high IFNG group UP) and *CXCR4* (Dupi DOWN, high IFNG group UP) Despite the regulation of AD-related driver genes, a large number of DEGs were not included in the dupilumab response gene signature for both the low (70.4%) and high (72.2%) IFNG AD groups (Figure 6C). Interestingly, within the genes exclusively regulated in the high IFNG AD group (507 genes), IFNG response genes such as *GATA3*, *GBP5*, *GBP1*, *GZMA*, *IFNG*, *IL32*, *STAT4*, *TNF*, *TRPV2* and *ZBP1* were highly upregulated (Supplementary Tables S1 and S2).

In summary, a strong common signature was observed between the high and low IFNG AD subgroups and the dupilumab response genes, including several AD driver genes that were regulated in opposite directions after the dupilumab treatment. However, the genes exclusively regulated in the high IFNG AD group were strongly associated with type 1 inflammation and were not affected by the dupilumab treatment.

## 3. Discussion

This study identified high and low IFNG AD subgroups by transcriptome and T cell secretome analyses, characterized their differences, and further evaluated their relevance for dupilumab treatments at the transcriptome level. Due to the significant biological role of IFN-γ in AD, such as in antiviral defense, this study focused on IFN-γ subgroups. Additionally, IFN-γ signaling pathways are often masked in unsupervised clustering algorithms due to strong type 2 responses. Given the diverse therapeutic landscape for AD, ranging from targeted therapies to JAK inhibition, selecting the appropriate treatment for each patient is crucial for physicians. While targeted therapies block type 2 cytokines, such as IL-13, JAK inhibitors block multiple cytokines, including IFN-γ. Understanding the molecular characteristics of high and low IFN-γ subgroups could provide a basis for future studies to stratify patients for either JAK inhibition or targeted therapy. First, mapping IFNG subgroups with their clinical parameters revealed that the high IFNG AD group was characterized by a higher age, a higher risk of cardiovascular comorbidities, and an intrinsic AD phenotype, while the low IFNG AD group showed classical features of extrinsic AD with atopic comorbidities and a downregulation of lipid metabolism. These findings are consistent with previous studies on acute/chronic and intrinsic/extrinsic AD [35,36,37]. Additionally, in line with our findings in the high IFNG AD group, Th1/type 1-associated cytokines were identified in the adult- compared to the pediatric-onset AD cohort, in chronic compared to acute AD, and in intrinsic compared to extrinsic AD [38,39,40,41]. Furthermore, the PCA of the low IFNG AD group revealed less separation between the lesional and non-lesional transcriptome in comparison to the high IFNG group, which might be explained by a strong alteration of healthy skin in extrinsic AD [42,43,44]. Moreover, in the low IFNG AD group, a significant reduction was observed in lipid production (glycerolipid metabolic process), a hallmark of the impaired barrier characteristic of extrinsic AD [41,45,46].

The protective function of IFN-γ in AD is an established mechanism in the context of its antiviral and antibacterial response [24,35,47]. This is supported by our data, as in the high IFNG AD group the pathways response to bacterium and positive regulation of defense response were significantly upregulated. This might explain why patients with intrinsic AD suffer less frequently from eczema herpeticum or generalized impetiginization (Staph. aureus) than patients with extrinsic AD [48,49]. Additionally, the pathway analysis of the high IFNG AD group also provides evidence for a pronounced proinflammatory milieu with an upregulation of the innate immune response, lymphocyte activation, response to cytokines and inflammatory response, as well as a strong activation of the MADAD and immune system gene sets. In this context, cytokine synergisms between IFN-γ and other cytokines may play a role. IFN-γ and TNF, for instance, exert pro-apoptotic effects on keratinocytes, which can contribute to eczema formation, cell death and spongiosis [50,51]. However, the transcriptome analysis of the high IFNG group revealed, overall, a stronger Th1 signal. As AD is a heterogenous type 2-dominant disease, it is important to understand the characteristics of patients exhibiting additional type 1 immune activation.

In contrast to previous studies, the present work compared the cellular composition of the high and low IFNG subgroups using digital cytometry (CIBERSORTx). Here, the high IFNG AD group showed higher estimated fractions for M1 macrophages and a trend towards higher levels of NK cells. Overall, the digital cytometry results revealed only minor statistical effects. This is expected, as lesional skin was compared with lesional skin, rather than comparing lesional skin with healthy skin. However, M1 macrophages revealed a significant difference between the two groups. While the focus of AD research is on the adaptive immune system, comparatively little is known about the role of M1 macrophages in AD [52]. M1 macrophages exhibit pro-inflammatory effects, as they release the cytokines IL-1β, IL-6, IL-12, IL-23, iNOS, MCP-1 and TNF-α, while M2 macrophages tend to have an anti-inflammatory and wound-healing-promoting effect through the production of IL-10, VEGF and TGF-β [53,54]. M2 macrophages, on the other hand, are the dominant type in allergic diseases such as bronchial asthma [53,55]. In general, the polarization of M1 macrophages is also induced by IFN-γ, GM-CSF and LPS in the course of a disturbed barrier [56]. In terms of AD, an increased amount of macrophages have been observed in acute and chronic AD lesions compared to non-lesional and healthy skin [52,57].

Additionally, in an AD mouse model, a similar M1/M2 ratio was observed in skin lesions; however, no classification into phenotypes or endotypes was evaluated [58]. The continuous M1 polarization in the high IFNG AD subgroup might promote a chronic inflammatory state [53]. Nevertheless, those results were derived from a mathematical approach and validation experiments are needed to confirm these findings. The relevance of IFN-γ-defined AD subgroups for treatment decisions is an intriguing but unanswered question. In our data, both subgroups share a strong Th2/type 2 signature. While the expression of *IL4RA* and *IL13RA1* correlates more with the low IFNG AD cohort, their comparison with the dupilumab response genes shows a great overlap between both groups. This observation is in line with a recent publication demonstrating the consistent efficacy of dupilumab in intrinsic and extrinsic AD [59]. However, whether high and low IFNG subgroups represent stable endotypes remains uncertain and can only be conclusively addressed through prospective studies assessing treatment responses. Our study has some limitations. First, due to a lack of publicly available gene expression datasets during JAK inhibition, no comparison with a JAK inhibitor response signature could be performed. Second, further clinical information on superinfections and disease duration was not available. While the results of the digital cytometry analysis revealed significant differences in M1 macrophages, there has been no protocol established to directly isolate this cell type from a skin biopsy and validate those findings experimentally. However, the strength of this study lies in its large transcriptome dataset, from 48 patients, of lesional and autologous non-lesional biopsies, combined with extensive clinical parameters. Classifying IFNG subgroups using the elbow method is a robust and novel methodology and provides a solid foundation for future therapeutic response evaluations in large AD transcriptome cohorts.

In clinical practice, treatment decisions require a careful evaluation of the individual effect/side-effect ratio. The long-term observation of tofacitinib, a pan-JAK inhibitor, in rheumatoid arthritis patients showed an increased risk of cardiovascular events and malignancies in patients over 50 years [60]. In general, the overall cardiovascular risk in AD patients is still controversial. On the one hand a meta-analysis indicated an increased risk of CVD correlating with AD disease severity [61]; on the other hand, the absolute attributable risk for CVD associated with AD was shown to be rather low and is associated with age and comorbidities, such as smoking [62,63]. However, recent short-term evaluations of JAK inhibitors in AD do not show an increased risk of cardiovascular events [64], with caveats such as the fact that long-term data for dermatology are currently not available. Thus, in this study, the high IFNG AD group showed a higher average age, suffered more frequently from arterial hypertension and had an upregulation of the pathway for cardiovascular disease. These results may suggest that the high IFNG subgroup may require further investigation regarding cardiovascular comorbidities and adverse events associated with JAKi in elderly patients. Despite these concerns, our data demonstrate that both groups exhibit a strong dupilumab response signature, indicating that targeting type 2 cytokines is a safe and effective approach for both subgroups. Future research might identify other modes of action targeting both type 2 cytokines and IFN-γ with a better risk–benefit profile in elderly patients. To the best of our knowledge, this is the first transcriptome analysis investigating high and low IFNG groups in an AD cohort using lesional and autologous non-lesional skin specimens from 48 patients.

In summary, this study provides further insights into the heterogeneity of AD and sheds light on the complexity of the immune pathways of AD subgroups. Differences in the clinical characteristics and the cellular composition of the high and low IFNG AD subgroups warrant future research on additional immune pathways, such as type 1-related pathways, involved in AD pathogenesis aside from type 2 immunity. Through a detailed characterization of individual immune deviations, a personalized approach to treatment decisions might allow for better response outcomes in the future.

## 4. Materials and Methods

### 4.1. Study Cohort

The transcriptome analysis of the lesional and non-lesional skin of 48 patients with AD (male *n* = 33, female *n* = 15) is a sub-analysis derived from the transcriptome project “IMCIS” (Head: Professor Kilian Eyerich) from the Biobank Biederstein [65]. The study design and data protection rules were approved by a local ethics committee (Klinikum Rechts der Isar, 44/16 S, 5590/12). The study was designed in line with the guidelines of the Declaration of Helsinki. After obtaining informed consent from all patients, clinical, histological and laboratory parameters were evaluated. Additionally, punch biopsies (6 mm) from the lesional and non-lesional skin of each patient were collected. One-third of the biopsies were used for RNA analysis, one-third for histological analysis and one-third for the isolation of lesional T cells.

### 4.2. RNAseq Library Preparation, Sequencing, Mapping and Quantification

Samples were procured and aligned as described previously [65]. In summary, first, the isolation of RNA from skin biopsies, using the QIAzol Lysis Reagent (Qiagen, Venlo, The Netherlands) and the miRNeasy Mini Kit (Qiagen), was performed. Next, RNASeq libraries were prepared using the TruSeq Stranded Total RNA Kit (Illumina, San Diego, CA, USA) according to the manufacturer’s protocol for high sample volumes. Finally, samples were sequenced on an Illumina HiSeq4000 (paired-end, read length of 2 × 150 bp, average output of 40 million reads per sample). The STAR Aligner was used for sequence alignment using the human genome reference hg38. The RNAseq count data were normalized by the median of ratios method, implemented in the DESeq2 protocol, and then transformed using the variance-stabilizing transformation from the Bioconductor (https://bioconductor.org/, 27 May 2024) package DESeq2 (https://bioconductor.org/packages/release/bioc/html/DESeq2.html, 28 May 2024).

### 4.3. Cluster Analysis (Elbow Method, K-Mean Clustering) and RNAseq Analysis

For the cluster analysis, normalized counts of IFNG expression in lesional skin samples were obtained, which were previously generated using the R (version 4.2.1) package DESeq2 [66] with respect to the condition lesional (*n* = 46) vs. non-lesional (*n* = 46). Throughout the analysis, the dataset was corrected for both gender and batch effects, executed through a design function in the DESEQ2 protocol. In alignment with the gender correction protocol, all Y-chromosome-associated genes were initially excluded. To determine the optimal number of clusters regarding lesional IFNG expression, the elbow method was applied in Python 3.7, employing the sklearn (https://scikit-learn.org/, 28 May 2024) and matplotlib (https://matplotlib.org/, 28 May 2024) packages. Subsequently, unsupervised k-means clustering was performed for the optimal number of clusters, determined via the elbow method in R (Version 4.2.1). Next, a re-analysis of the AD cohort was performed according to the DESeq2 protocol from Bioconductor, as described above, and the AD cohort was divided based on the groups established by the k-means clustering results, contrasting the lesional and non-lesional samples and comparing the lesional transcriptomes of the high and low IFNG AD groups. Subsequently, a gene set enrichment analysis was conducted in R using the clusterProfiler package (https://bioconductor.org/packages/release/bioc/html/clusterProfiler.html, 28 May 2024), org.Hs.eg.db (https://bioconductor.org/packages/release/data/annotation/html/org.Hs.eg.db.html, 28 May 2024) and the Gene Ontology (GO) database (https://geneontology.org/, 28 May 2024). The criteria for significance were stringent, setting cut-offs at a fold change (FC) of +/−1 and a false discovery rate (FDR) less than or equal to 0.05.

### 4.4. Digital Cytometry/Deconvolution—CIBERSORTx

For the immune phenotyping of the bulk RNA sequencing data, a deconvolution algorithm was run on the CIBERSORTx platform (https://cibersortx.stanford.edu/, 28 May 2024), as previously described [67]. LM22, a gene matrix of 22 distinct human hematopoietic cell populations, was used as the signature matrix [68]. The analysis was performed for the parameters’ batch correction in B-mode (comparing RNASeq with microarray data) and quantile normalization. The deconvolution algorithm was performed with 50 permutations.

### 4.5. Gene Set Variation Analysis

A gene set variation analysis (GSVA) was performed in R (4.2.1) as part of the bioconductor package (version 3.18), as previously described [69], on the counts per million (CPM) data of the whole AD cohort (lesional vs. non lesional data of all 46 patients). Subsequently, the results were displayed for lesional (Disease), non-lesional (Healthy), Cluster 3 (high IFNG AD) and Cluster 1 (low IFNG AD) samples. The publicly available gene set from the meta-analysis-derived atopic dermatitis (MADAD) study [27], as well as gene sets for cardiovascular/artherosclerosis-associated genes [28,29,30,70] and immune associated genes [29,31], were analyzed. For the analysis of type 1 and 2 keratinocyte response genes, 2D keratinocytes were stimulated with IFN-γ (20 ng/mL), for type 1, and IL-13 (20 ng/mL), for type 2 conditions, for 16 h and whole-genome expression arrays (SurePrint G3 Human GE 8X60K BeadChip (#G4858A-028004, Agilent Technologies, Santa Clara, California, CA, USA) were performed according to the manufacturer’s instructions, as described previously [32]. Cut-offs were set as a *p*-value ≤ 0.05 and fold change ≥ 1.

### 4.6. Inform Algorithms and Comparison with Dupilumab Signature

The identification of the most important co-expressed genes in the high and low IFNG AD groups was performed, as previously described, with the INfORM software (https://github.com/Greco-Lab/INfORM, 28 May 2024) [34], using FKPMs of the DEGs as the data background. The settings were left as default. As also previously described, the algorithm was performed without mrnetb to reduce computation time [71]. To compare the top co-expressed genes with the dupilumab signature, RNA sequencing data from Wk16 (16 weeks after initiation of dupilumab treatment) vs. Wk0 (the baseline transcriptome before initiating dupilumab treatment), derived from Guttman-Yassky et al., were filtered for FDR ≤ 0.05 and |log2FC| ≥ 1 [33] and compared to the co-expressed gene network of the high and low IFNG DEGs via the online Venny 2.1.0 tool (https://bioinfogp.cnb.csic.es/tools/venny/, 28 May 2024).

### 4.7. Visualization

The data were visualized in GraphPadPrism9, in R (version 4.2.1), using the packages ggplot2 (https://ggplot2.tidyverse.org/, 28 May 2024), enrichplot (https://www.bioconductor.org/packages/release/bioc/html/enrichplot.html, 28 May 2024), DOSE (https://www.bioconductor.org/packages/release/bioc/html/DOSE.html, 28 May 2024), EnhancedVolcano (https://bioconductor.org/packages/release/bioc/html/EnhancedVolcano.html, 28 May 2024), cnetplot (https://rdrr.io/bioc/enrichplot/man/cnetplot.html, 28 May 2024) and ggforty (https://cran.r-project.org/web/packages/ggfortify/index.html, 28 May 2024); in Biorender (license included https://www.biorender.com/, 28 May 2024); in Python (3.7), with the package Matplotlib; and in Adobe Illustrator (Version 28.5).

### 4.8. Analysis of the Secretome of Lesional T Cells

Primary human lesional T cells were isolated from fresh skin biopsies of patients with AD (*n* = 7) by their migration towards an IL-2 gradient, as described previously [72,73,74]. Briefly, biopsies were incubated in T cell proliferation medium (RPMI 1640 medium containing 5% human serum (Sigma-Aldrich, St. Louis, MO, USA), 2 mmol/L glutamine, 1 mmol/L sodium pyruvate, 1% non-essential amino acids, 100 U/mL penicillin and 100 mg/mL streptomycin (all reagents from Invitrogen, Carlsbad, CA, USA) supplemented with 60 U/mL IL-2 (Proleukin, Novartis, Basel, Switzerland) until the lesional T cells that had emigrated from the biopsy reached a confluence of 30–50%. The T cell proliferation medium, supplemented with IL-2, was replenished three times a week. After the isolation phase, emigrated lesional T cells were expanded using α-CD3 (0.75 µg/mL pre-coated, BD Biosciences) and α-CD28 (0.75 µg/mL, soluble, BD Biosciences, Franklin Lakes, NJ, USA) stimulation. For supernatant generation, cells were re-stimulated with α-CD3/α-CD28 for 3 days and the supernatant was characterized by an enzyme-linked immunosorbent assay (ELISA) for IL-4 (BD, 555194), IFN-γ (R&D Systems, DY285B, Minneapolis, MN, USA) and IL-22 (R&D Systems, DY782) according to the manufacturer’s recommendations. Supernatants from patients 6–7 and from patients 1–5 were pooled in equimolar ratios and their protein levels were analyzed by a multiplex ELISA, using the Pro Human Cytokine 27-plex Assay (Bio-Rad Laboratories, Hercules, CA, USA) according to the manufacturer’s recommendation.

### 4.9. Language Editing

Language editing, grammar correction and translation tasks were performed with the assistance of DeepL, ChatGPT4 and Grammarly. All analyses and text content in this manuscript originate from the authors and were not created by AI.

### 4.10. Statistical Analysis

For the statistical analysis of the deconvolution proportion between the high and low IFNG AD groups (Figure 4), in terms of age (Figure 1) and gene expression (Figure 3), a paired *t*-test was performed. For the age analysis of Figure S1, a one-way ANOVA test was used. Values were displayed as mean ± standard error of mean (SEM). The analysis between the Z-scores of the different clusters in the GSVA was conducted using a mixed-effect analysis with Tukey’s multiple comparison test. The significance levels were defined as *p* ≤ 0.05 (*), *p* ≤ 0.01 (**), *p* ≤ 0.001 (***) and *p* ≤ 0.0001 (****).

## Data Availability

The datasets related to this article can be found at GEO under the accession number GSE154200 in an open-source online data repository hosted at NCBI.

## Supplementary Materials

The following supporting information can be downloaded at https://www.mdpi.com/article/10.3390/ijms25116158/s1.
