# Supplementary material for: Characterization of High and Low IFNG-Expressing Subgroups in Atopic Dermatitis
Source: Int J Mol Sci. 2024 Jun 3;25(11):6158. doi: 10.3390/ijms25116158 (PMC11173096; doi:10.3390/ijms25116158)
Supplement: Supplementary file 1 [file ijms-25-06158-s001.zip › Supplements_IFNG_AD_upload/Suppl_Fig_S2.pdf]

Figure S2

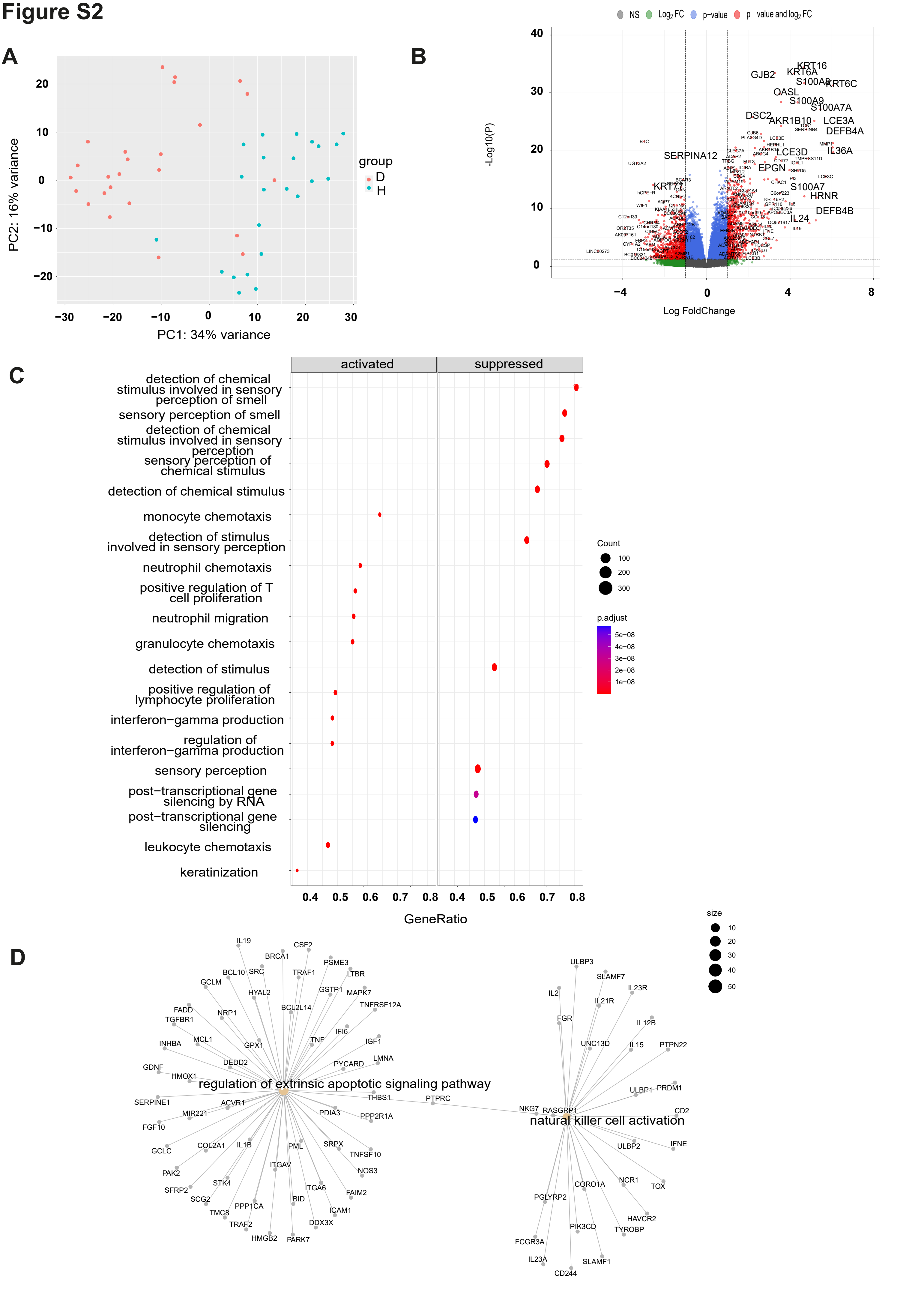

Figure S2. Transcriptome analysis of lesional versus non-lesional skin of the medium IFNG AD group.
